# ERBB-2 overexpression confers PI 3 **′** kinase-dependent invasion capacity on human mammary epithelial cells

**DOI:** 10.1054/bjoc.1999.0979

**Published:** 2000-01-18

**Authors:** K M Woods Ignatoski, T Maehama, S M Markwart, J E Dixon, D L Livant, S P Ethier

**Affiliations:** Departments of Radiation Oncology, Biological Chemistry and Anatomy and Cell Biology, University of Michigan Medical School, Ann Arbor, MI, 48109–0616, USA

**Keywords:** ERBB-2, invasion, P13′ kinase, human breast cancer, human mammary epithelial cells

## Abstract

Amplification and overexpression of ERBB-2 in human breast cancer is thought to play a significant role in the progression of the disease; however, its precise role in the aetiology of altered phenotypes associated with human breast cancer is unknown. We have previously shown that exogenous overexpression of ERBB-2 conferred growth factor independence on human mammary epithelial cells. In this study, we show that ERBB-2 overexpression also causes the cells to acquire other characteristics exhibited by human breast cancer cells, such as anchorage-independent growth and invasion capabilities. ERBB-2-induced invasion is dependent on fibronectin and correlates with the down-regulation of cell surface α4 integrin. In addition ERBB-2 co-immunoprecipitates with focal adhesion kinase (FAK) in these cells. We have also shown, by use of exogenously expressed PTEN and by treatment with the PI3′-kinase inhibitor LY294002, that ERBB-2-induced invasion is dependent on the PI3′-kinase pathway; however, PTEN does not dephosphorylate FAK in these cells. © 2000 Cancer Research Campaign


*British Journal of Cancer* (2000) **82**(3), 666–674
© 2000 Cancer Research Campaign
DOI: 10.1054/ bjoc.1999.0979, available online at http://www.idealibrary.com on IDEAL®

### ERBB-2 overexpression confers PI 3′ kinase-dependent invasion capacity on human mammary epithelial cells

**KM Woods Ignatoski[1], T Maehama[2], SM Markwart[3], JE Dixon[2], DL Livant[3] and SP Ethier[1]**

Departments of [1]Radiation Oncology, [2]Biological Chemistry and [3]Anatomy and Cell Biology, University of Michigan Medical School, Ann Arbor, MI 48109–0616, USA

**Summary** Amplification and overexpression of ERBB-2 in human breast cancer is thought to play a significant role in the progression of the disease; however, its precise role in the aetiology of altered phenotypes associated with human breast cancer is unknown. We have previously shown that exogenous overexpression of ERBB-2 conferred growth factor independence on human mammary epithelial cells. In this study, we show that ERBB-2 overexpression also causes the cells to acquire other characteristics exhibited by human breast cancer cells, such as anchorage-independent growth and invasion capabilities. ERBB-2-induced invasion is dependent on fibronectin and correlates with the down-regulation of cell surface α4 integrin. In addition ERBB-2 co-immunoprecipitates with focal adhesion kinase (FAK) in these cells. We have also shown, by use of exogenously expressed PTEN and by treatment with the PI3′-kinase inhibitor LY294002, that ERBB-2-induced invasion is dependent on the PI3′-kinase pathway; however, PTEN does not dephosphorylate FAK in these cells. © 2000 Cancer Research Campaign

**Keywords:** ERBB-2; invasion; P13′ kinase; human breast cancer; human mammary epithelial cells

Amplification and overexpression of genes that regulate growth and differentiation of normal cells is a common mechanism for oncogene activation in human solid cancers. Among the oncogenes known to be amplified and overexpressed in human breast cancer (HBC) are ERBB-2 (Kraus et al, 1987; Slamon et al, 1987; Berger et al, 1988; Guerin et al, 1989) (also known as HER-2/neu), C-MYC (Bonilla et al, 1988; Borg et al, 1992; Watson et al, 1993), CCDN1 (Gillet et al, 1994), FGFR 1, 2 and 4 (Luqmani et al, 1992; Jaakkola et al, 1993; Jacquemier et al, 1994), as well as other less well characterized genes (Bieche et al, 1996; Anzick et al, 1997; Sen et al, 1997). In addition to gene amplification, HBC cells often acquire other genetic alterations that may play a role in altered phenotypes expressed by cancer cells. And, while it is often possible to determine the signalling pathways activated as a result of gene amplification, it is much more difficult to discern the role of a specific genetic change in the expression of altered growth phenotypes exhibited by breast cancer cells.

Previously, we demonstrated that HBC cells with an amplification of ERBB-2 express high levels of constitutively activated $p185^{ERBB-2}$ and exhibit varying degrees of growth factor independence in vitro that is directly related to the level of overexpression of the gene (Ram et al, 1996). More recently, we have been able to recapitulate the growth factor independence phenotype in immortalized human mammary epithelial (HME) cells by overexpressing ERBB-2 to levels equivalent to that expressed by breast cancer cells, indicating that ERBB-2 itself can drive factor independent growth when expressed to high enough levels (Woods Ignatoski et al, 1999). In the present studies, we investigated the role of ERBB-2 overexpression in the acquisition of other altered phenotypes exhibited by breast cancer cells. We found that high level overexpression of ERBB-2 in the immortalized HME cell line H16N2 results, not only in the acquisition of anchorage-independent growth capacity, but also in the ability to invade naturally occurring basement membranes in a manner similar to breast cancer cells. The ability of ERBB-2 to induce invasion is linked to its ability to activate PI 3′-kinase, involves down-regulation of $\alpha_4\beta_1$ integrins, and may involve signalling from focal adhesion kinase, which is associated with ERBB-2 in the overexpressing cells. Thus, when expressed to very high levels, ERBB-2 becomes associated with signalling complexes not detectable in normal cells, and is directly involved in the acquisition of a number of phenotypes characteristic of transformed cells.

*Received 2 June 1999*
*Revised 29 July 1999*
*Accepted 5 August 1999*

*Correspondence to:* SP Ethier

### MATERIALS AND METHODS

### Cell culture

The base medium for H16N2-PTP, H16N2-ERBB2 and SUM-190PT cells was Ham's F12 media supplemented with 0.1% bovine serum albumin, 0.5 μg ml$^{-1}$ fungizone, 5 μg ml$^{-1}$ gentamycin, 5 mM ethanolamine, 10 mM HEPES, 5 μg ml$^{-1}$ transferrin, 10 μM $T_3$, 50 μM selenium and 1 μg ml$^{-1}$ hydrocortisone. SUM-190PT cell medium was further supplemented with 5 μg ml$^{-1}$ insulin, and H16N2-PTP cell medium was further supplemented with 10 ng ml$^{-1}$ epidermal growth factor (EGF). The medium for SUM-52PE, SUM-149PT, and SUM-225CWN was Ham's F12 supplemented with 5% fetal bovine serum (FBS), 0.5 μg ml$^{-1}$ fungizone, 5 μg ml$^{-1}$ gentamycin and 5 μg ml$^{-1}$ insulin and further supplemented with 1 μg ml$^{-1}$ hydrocortisone. SkBr3 cells were grown in Dulbecco's modified Eagles medium (DMEM) supplemented with 10% FBS, 0.5 μg ml$^{-1}$ fungizone and 5 μg ml$^{-1}$ gentamycin. Cells infected with retroviral expression vectors were selected in 100 μg ml$^{-1}$ geneticin (G418) for 2 weeks. All cell culture reagents were obtained from Sigma Chemical Co. (St Louis, MO, USA). Detailed descriptions of all of the SUM cell lines can be found at http://p53.cancer.med.umich.edu/clines/clines.html on the world-wide web.

### Retrovirus construction pTPerbB-2 retrovirus construction was previously described (Harris et al, 1998; Woods Ignatoski et al, 1999). pTPPTEN(wt) and pTPPTEN (C124S) were constructed by excising the FLAG-tagged PTEN genes from FLAG-PTEN/CMV5 (Maehama and Dixon, 1998) with *Eco*RI and *Bam*HI. The ends of each PTEN gene were blunted and ligated into pTP 2000 which was digested with *Xho*I and blunt ended. Five micrograms of DNA were transfected into ϕNX-A cells by $Ca_2PO_4$ precipitation and virus was collected 48 h after transfection. Cells were infected with the appropriate virus and selected in 100 μg $ml^{-1}$ gentamycin for 2 weeks. Because erbB-2 overexpressing cells already contained a construct that was G418-resistant, we tested the quality of infectious pTPPTEN virus by infecting non-G418-resistant MCF-10A cells and selecting in 100 μg $ml^{-1}$ gentamycin for 2 weeks. Each pTPPTEN virus infected approximately 75% of the MCF-10A cells.

### Soft agar assays

Six-well dishes were coated with a 1:1 mix of the appropriate 2 × medium for the cell line being studied and 1% Bactoagar. Cells were plated at $1 \times 10^3$, $1 \times 10^4$ and $1 \times 10^5$ cells per well in a mixture of appropriate medium and 0.3% Bactoagar. Cells were fed 3 times per week for 3 weeks, stained with 500 μg $ml^{-1}$ P=R*ho* iodonitrotetrazolium violet (Catalog no. I-8377, Sigma Chemical Co., St Louis, MO, USA) overnight and photographed. Each cell line was plated in duplicate in three separate dilutions. The experiment was repeated three times.

### FAK immunoprecipitations and protein blots

Cells were lysed in a buffer containing 20 mM Tris–HCl, pH 8.0, 137 mM sodium chloride (NaCl), 1% NP-40, 10% glycerol, 1 mM $Na_3VO_4$, 1 mM phenylmethyl sulphoxide (PMSF), 1% aprotinin, 20 μg $ml^{-1}$ leupeptin. Protein concentrations were equalized to 100 μg for whole cell lysates or to 1 mg for immunoprecipitations using the Løwry method (Bradford, 1976). For whole cell lysates, Laemmli sample buffer (Sambrook et al, 1989) was added and the samples were boiled. For immunoprecipitations, 1 μg of α-FAK antibody (Catalog no. sc-558, Santa Cruz Biotechnology, Inc., Santa Cruz, CA, USA) was added to equivalent protein amounts of sample and incubated at 4°C for 1 h. Immune complexes were then bound to protein A/G beads for 1 h at 4°C. Immunoprecipitates were washed once in lysis buffer, twice in phosphate-buffered saline (PBS) containing 1% Triton X-100 and twice in PBS. Laemmli sample buffer was added and the samples were boiled. Equal amounts of protein were separated in 7.5% sodium dodecyl sulphate polyacrylamide gel electrophoresis (SDS-PAGE). Proteins were blotted to polyvinylidene difluoride (PVDF) membrane and probed with α-$Ptyr_{4G10}$ antibody (Catalog no. 05-321, Upstate Biotechnology, Inc., Lake Placid, NY, USA) or with α-FAK antibody. These experiments were repeated three times.

### PTEN immunoprecipitates and protein blots

Infected cells were lysed in a buffer containing 10 mM Tris–HCl, pH 8.0, 50 mM NaCl, 5 mM EDTA, 50 mM sodium fluoride (NaF), 1% NP-40, 1 mM $Na_3VO_4$, 1 mM PMSF. Anti-FLAG M2 beads (Catalog no. A-1205, Sigma Chemical Co.) were added to lysates and the mix was incubated for 2 h at 4°C. Immunoprecipitates were washed 3 × with lysis buffer and once with Tris-buffered saline (TBS)-0.03% Brij-35. Proteins were separated in 10% SDS-PAGE and blotted to PVDF membrane. Blots were probed with α-PTEN antibody (Catalog no. 06-894, Upstate Biotechnology, Inc.). The experiment was repeated twice.

### FACs analysis

Cells were removed from tissue culture plastic with a room temperature incubation with 10 mM EDTA in Hank's balanced salt solution, washed twice in PBS, then incubated with anti-integrin $\alpha_4$ antibody (Catalog no. 12077-012, Gibco) or with anti-integrin $\alpha_5$ antibody (Catalog no. CP12L, Oncogene Research Products, Cambridge, MA, USA) for 1 h at 22°C. Cells were then washed twice in PBS and incubated with a fluorescein isothiocyanate (FITC)-conjugated secondary antibody for 1 h at 37°C. After two washes in PBS, cells were fixed with 70% ethanol and subjected to fluorescence activated cell sorting.

### Invasion

Cells were suspended in 0.23% trypsin–EDTA (Catalog no. 15050–057, Gibco Life Technologies, Grand Island, NY) and placed on SU-basement membranes with or without fetal calf serum (FCS) according to established methods (Livant et al, 1995) for 4 h at 37°C, the time required to observe maximal invasion percentages for normal and metastatic cells (Livant et al, 1995, and data not shown). The percentages of spread and adherent cells were evaluated in each assay to check viability prior to fixation in 2% formaldehyde and scored at 400 × magnification using phase contrast optics. Viability ranged from 90 to 98% in all assays. Mean invasion percentages resulted from three independent determinations involving the scoring of all cells in contact with the invasion substrates. Fn-FCS was prepared by repeated affinity chromatography of FCS on gelatin sepharose as previously described (Catalog no. 17095601, Pharmacia Biotech, Piscataway, NJ, USA) (Akiyama and Yamada, 1985). The pFn depletion was confirmed by immunoblotting with α-Fn antibody (Catalog no. 12112–017, Gibco Life Technologies) (Hayman et al., 1982, data not shown). LY294002 (Catalog no. L-9908, Sigma Chemical Co.) stock solution was 25 mM in 100% ethanol. LY294002 was added to fresh cell media to a final concentration of 25 μM for 24 h prior to the start of the invasion assay. Ten microlitres of 100% ethanol was added to an identical plate as a control.

### RESULTS

### ErbB-2 overexpression results in anchorage-independent survival and growth of HME cells

In previous work, we developed a series of cell lines by retroviral infection of the immortalized HME cell line, H16N2, with a vector

© 2000 Cancer Research Campaign

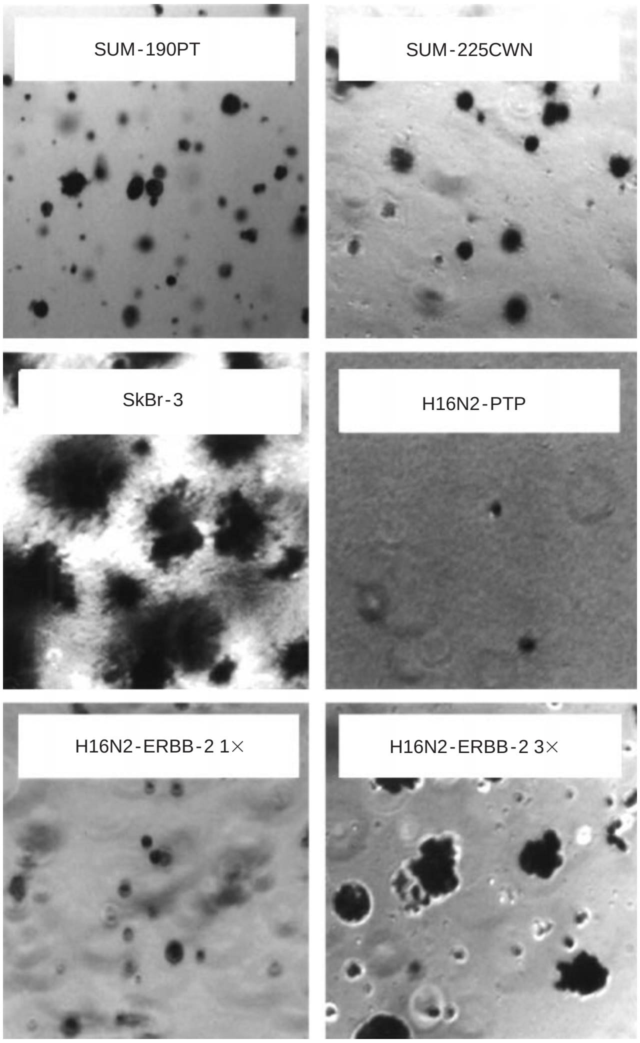

**Figure 1** Cells with ERBB-2 overexpression grow in an anchorage-independent manner. Control cells (H16N2/PTP), HBC cell lines (SUM-190PT, SUM-225CWN, and SkBr-3), and cells increasingly overexpressing ERBB-2 (1 × and 3 ×) were plated in agar, grown for 2 weeks, stained with a vital stain and photographed containing the full-length human ERBB-2 gene. We found that, when selected for proliferation in serum-free, growth factor-free medium, the transduced cells expressed very high levels of $p185^{ERBB-2}$ that were constitutively tyrosine phosphorylated. Importantly, these cells expressed levels of activated $p185^{ERBB-2}$ that are equivalent to levels expressed by HBC with an ERBB-2 gene amplification. Thus, as predicted from studies using ERBB-2 amplified HBC cells (Ram et al, 1996), high level ERBB-2 over-expression and activation results in multiple growth factor independence (Woods Ignatoski et al, 1999).

To begin to examine other altered phenotypes that result from ERBB-2 overexpression, H16N2 cells expressing different levels of ERBB-2 protein (infected 1 × with pTPERBB2 or infected 3 × with pTPERBB2-infected) were tested for their ability to grow in soft agar. Figure 1 demonstrates that, like breast cancer cells with an ERBB-2 gene amplification, H16N2 cells overexpressing ERBB-2 have the ability to survive under anchorage-independent conditions. Furthermore, H16N2 cells expressing the highest levels of $p185^{ERBB-2}$ (3 × pTPERBB2-infected) grew to form large colonies in agar. Thus, progressive overexpression of ERBB-2 resulted in increasing ability of HME cells to grow in an anchorage-independent manner. These results are consistent with those reported previously by Pierce et al (1991).

### ERBB-2 overexpressing cells acquire invasive potential

To assess the invasive capacity of H16N2-ERBB2 cells, we used a novel assay that utilizes naturally occurring sea urchin (SU) embryo basement membranes. This experimental system allows one to assess the invasive capacity of human cancer cells and transformed cells in a completely defined system, because, as they are obtained from an invertebrate, SU-basement membranes are naturally serum-free. This technique has been described previously and accurately reflects the in vivo invasive and metastatic capacity of a variety of cancer and normal cell types (Livant et al, 1995).

We first assessed the invasive capacity of human breast cancer cell lines developed in our laboratory, and compared that to the invasive capacity of H16N2-ERBB2 cells, and vector transduced controls. Figure 2A shows that three human breast cancer cell lines, all of which were derived from aggressive breast cancers, but which have different molecular etiologies, expressed significant invasive capacity in this system. By contrast, HME cells failed to invade under these same conditions. H16N2-ERBB2 cells exhibited invasive capacity similar to that expressed by breast cancer cells, whereas control vector-transduced cells were not invasive. These results indicate that high level overexpression of $p185^{ERBB-2}$ results in important transformed phenotypes beyond growth factor independence and anchorage independent growth.

It was demonstrated previously that invasion of prostate cancer cells through SU-basement membranes is dependent on the presence of plasma fibronectin, and that the PHSRN sequence of the fibronectin cell-binding domain is sufficient to stimulate invasion (DL Livant, manuscript submitted). The PHSRN sequence is a known ligand for the $\alpha_5\beta_1$ integrin (Aota et al, 1994). It has been shown previously that $\alpha_5\beta_1$ integrin binding to plasma fibronectin fragments containing the cell-binding domain but not the C-terminal domain, which contains the binding site for $\alpha_4\beta_1$ integrin, results in the activation of metalloproteinase gene expression in fibroblasts which is essential for invasion (Huhtala et al, 1995). We therefore examined the dependence on plasma fibronectin for the invasion of breast cancer cells and H16N2-ERBB2 cells, as well as their expression of $\alpha_5\beta_1$ and $\alpha_4\beta_1$ integrins. Figure 2B shows that breast cancer cells and H16N2-ERBB2 cells were invasive in the presence of serum, but not serum that had been specifically depleted of fibronectin. Similarly, cells were only invasive in serum-free conditions when fibronectin was added to the medium.

To examine integrin expression in these cells, FACs analysis was performed using the cell lines described above following incubation of intact, non-fixed cells with fluorescently tagged antibodies against the $\alpha_4$ and $\alpha_5$ integrins. Table 1 shows that breast cancer cells express dramatically lower levels of cell surface $\alpha_4$ integrins than do normal HME cells, consistent with results obtained previously with prostate epithelial and invasive prostate cancer cells (Witkowski et al, 1993; Roklin and Cohen, 1995). Similarly, control vector transduced H16N2 cells, which are non-invasive, express $\alpha_4$; whereas, this integrin was down-regulated from the cell surface in invasive H16N2-ERBB2 cells.

 © 2000 Cancer Research Campaign

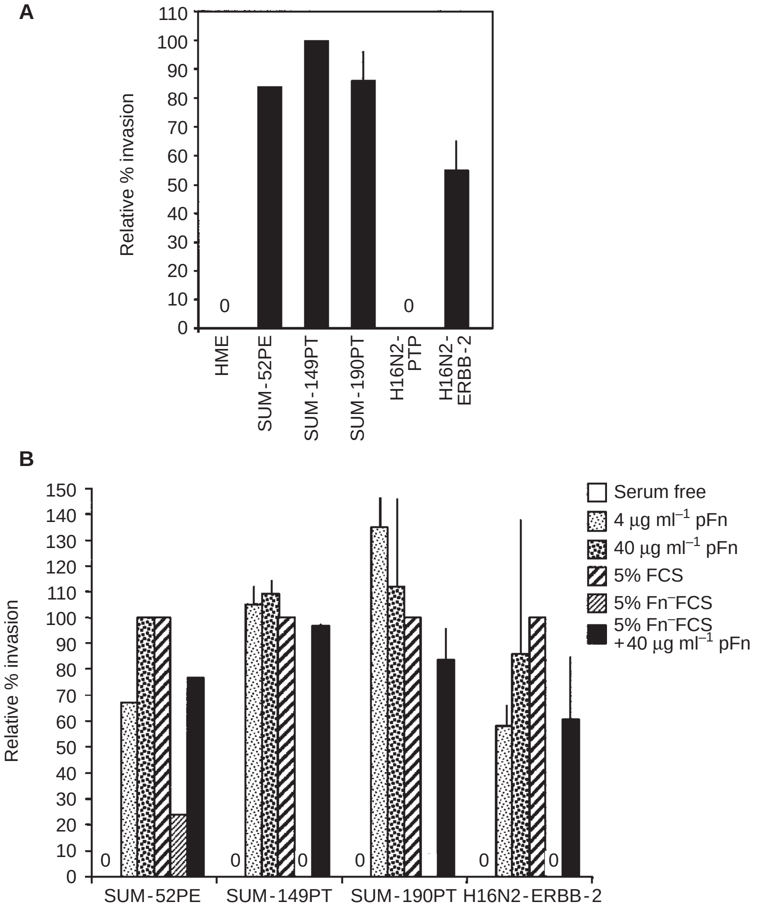

**Figure 2** HBC cells and H16N2-ERBB-2 cells undergo fibronectin-dependent invasion. (**A**) Invasion assays for HME, HBC, and H16N2-ERBB-2 cells. Percent invasion relative to SUM-149PT invasion given. (**B**) Invasion assays for HBC and H16N2-ERBB-2 cells were performed in serum-free medium or serum-free medium supplemented with FCS or pFN, as indicated. Per cent invasion relative to 5% FCS invasion was graphed

**Table 1** Cell surface integrin expression[a]

| Cell line | $\alpha_4$ expression[b] | $\alpha_5$ expression[b] |
|---|---|---|
| HME | 54 | 62 |
| SUM-149PT | 0 | 10 |
| SUM-190PT | 14 | 27 |
| H16N2-PTP | 55 | 47 |
| H16N2-ERBB-2 | 11 | 86 |
| H16N2-ERBB2/PTENWT | 12 | 21 |

[a]As determined by FACs analysis. [b]Per cent increase in amount of staining for $\alpha_4$ or $\alpha_5$ integrins over negative control.

Interestingly, $\alpha_5$ expression was increased in the invasive H16N2-ERBB2 cells as compared to the vector transduced controls. These results are consistent with the hypothesis that invasion by transformed breast epithelial cells through naturally occurring basement membranes is mediated by interactions between specific integrins and particular domains of plasma fibronectin. Thus, like invasive breast and prostate cancer cells, H16N2-ERBB2 cells have reduced expression of cell surface $\alpha_4\beta_1$ integrins, which bind to a specific region of plasma fibronectin, and which may be functionally relevant to their capacity to invade basement membranes. Experiments are underway to evaluate the role of cell surface $\alpha_4$ integrin in the invasive behaviour of HME cells.

### Signalling pathways mediating the invasive phenotype in ERBB-2 overexpressing cells

We and others have shown previously that an important consequence of ERBB-2 overexpression is a dramatic increase in the level of activation of phosphatidylinositol 3′ kinase (PI 3′ kinase) and its downstream signalling pathways (Fedi et al, 1994; Soltoff et al, 1994; Ram and Ethier, 1996). Therefore, a series of experiments were performed to assess the role of this pathway in the invasive potential of H16N2-ERBB2 cells.

The tumour suppressor gene, PTEN, is a phosphatase that regulates the activity of the PI 3′-kinase pathway by virtue of its ability to directly dephosphorylate PIP3 (Maehama and Dixon, 1998). Therefore, we prepared retroviral expression vectors containing full-length human PTEN, or a mutant PTEN that has the ability to bind substrate but which is defective in both its lipid and protein phosphatase activities. Figure 3A illustrates the constructs used, and Figure 3B shows the expression of the transduced PTEN proteins in H16N2-ERBB2 cells following infection with the

© 2000 Cancer Research Campaign 

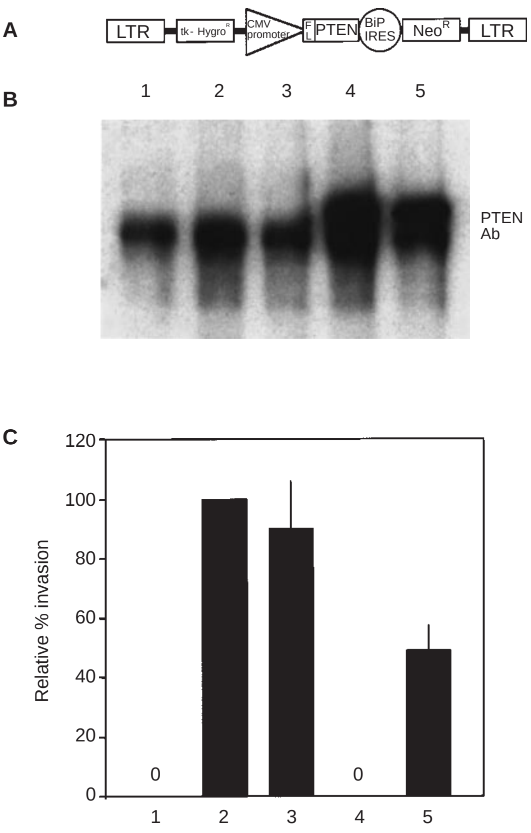

**Figure 3** PI3′ kinase is involved in the invasion of HBC and ERBB-2 overexpressing cells. (**A**) FLAG-tagged PTEN and PTEN CS (C124S) were ligated into the biscistronic vector pTP2000. FL = FLAG Tag. (**B**) Western blot on anti-FLAG immunoprecipitates using anti-FLAG antibody showing that H16N2-ERBB-2/PTEN and H16N2-ERBB-2/PTEN CS were expressing exogenous PTEN. Lanes: H16N2-PTP, 1; H16N2-ERBB-2, 2; H16N2-ERBB-2/PTP, 3; H16N2-ERBB-2/PTEN, 4; H16N2-ERBB-2/PTEN CS, 5. **(C)** H16N2-ERBB-2/PTEN WT and H16N2-ERBB-2/PTEN CS cells were used in invasion assays. Columns: H16N2-PTP, 1; H16N2-ERBB-2, 2; H16N2-ERBB-2/PTP, 3; H16N2-ERBB-2/PTEN, 4; H16N2-ERBB-2/PTEN CS, 5. Per cent invasion relative to H16N2-ERBB-2 invasion was graphed

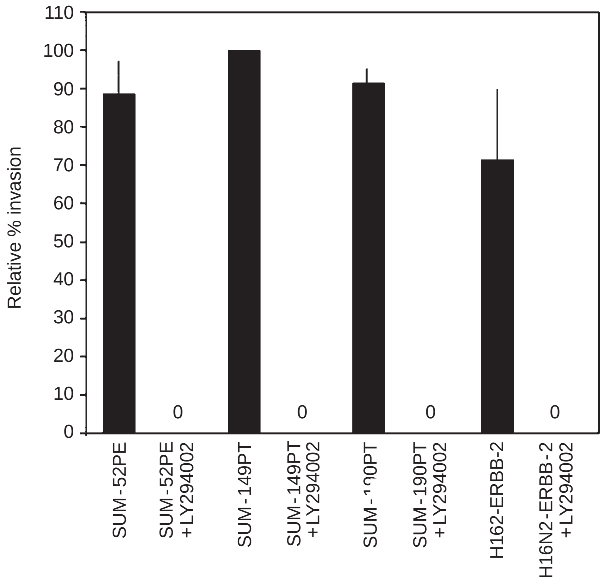

**Figure 4** The PI 3′-kinase inhibitor, LY294002, blocks invasion of HBC and ERBB-2 overexpressing cells. HBC cells and H16N2-ERBB-2 cells were incubated with LY294002 then tested for invasion. Per cent invasion relative to SUM-149PT invasion given retroviral vectors. Next, basement membrane invasion experiments were performed using H16N2-ERBB2 cells and PTEN-infected versions of the same cells. Figure 3C shows that, whereas H16N2-ERBB2 cells are invasive, overexpression of wild-type PTEN completely abrogated their invasive capacity. Infection of H16N2-ERBB2 cells with a vector containing the cs-PTEN mutant reduced, but did not eliminate, the invasive capacity of the cells. This result is consistent with the ability of the mutant protein to bind and sequester substrate without dephosphorylating it, which allows the mutant PTEN to partially block PIP3 signalling (Maehama and Dixon, 1998).

To confirm that signalling from PI 3′-kinase is important for ERBB-2-induced invasion of basement membranes, H16N2-ERBB2 cells and control cells were incubated with the PI 3′-kinase inhibitor LY294002 and tested for invasive capacity. Figure 4 shows that this compound, like PTEN, completely blocked the invasive capacity of H16N2-ERBB2 cells.

Recently, Tamura et al (1999) demonstrated that PTEN blocks the invasiveness of transformed cells by directly dephosphorylating $pp125^{FAK}$. However, the experiments of Maehama and Dixon (1998) suggests that, under conditions that are relevant to the in vivo situation, $pp125^{FAK}$ is a poor substrate for PTEN. Therefore, we performed experiments to assess the $pp125^{FAK}$ protein activation status in invasive H16N2-ERBB2 cells versus non-invasive control cells, and to assess the role of PTEN in modulating $pp125^{FAK}$ tyrosine phosphorylation in these cells.

To determine if the level of tyrosine phosphorylated $pp125^{FAK}$ was elevated in invasive H16N2-ERBB2 cells compared to controls, FAK immunoprecipitates were used to prepare Western blots that were probed with phosphotyrosine antibodies. The data in Figure 5A shows that overexpression of $p185^{ERBB-2}$ in H16N2-ERBB2 cells did not result in significant changes in the levels of tyrosine phosphorylated $pp125^{FAK}$. However, in these blots, we did detect a tyrosine phosphorylated p185 that co-immunoprecipitated with FAK in the ERBB-2 overexpressing cells, but not in control cells. Reprobing of an identical blot with ERBB-2 specific antibodies confirmed the identity of the tyrosine phosphorylated protein as $p185^{ERBB-2}$. The data in Figure 5A also shows that the association of $pp125^{FAK}$ with $p185^{ERBB-2}$, detected in the H16N2-ERBB-2 cells, is also detectable in the SkBr-3 breast cancer cell line which expresses high levels of $p185^{ERBB-2}$ as a result of a gene amplification. Thus, whereas levels of tyrosine phosphorylated FAK are not altered in invasive ERBB-2 overexpressing cells, $p185^{ERBB-2}$ can be detected in immunocomplexes with $pp125^{FAK}$ when ERBB-2 is expressed to high levels.

Experiments were next performed to examine the influence of PTEN overexpression on levels of tyrosine phosphorylated $pp125^{FAK}$ and its association with $p185^{ERBB-2}$. The results shown in Figure 5B indicate that PTEN overexpressing cells that have lost their invasive capacity express the same levels of tyrosine phosphorylated $pp125^{FAK}$ as non-PTEN overexpressing cells with full invasive capacity. Furthermore, PTEN expression did not influence the association of $p185^{ERBB-2}$ with $pp125^{FAK}$, and did not result in the re-expression of $\alpha_4$ integrin on the cell surface (Fig. 5 and

 © 2000 Cancer Research Campaign

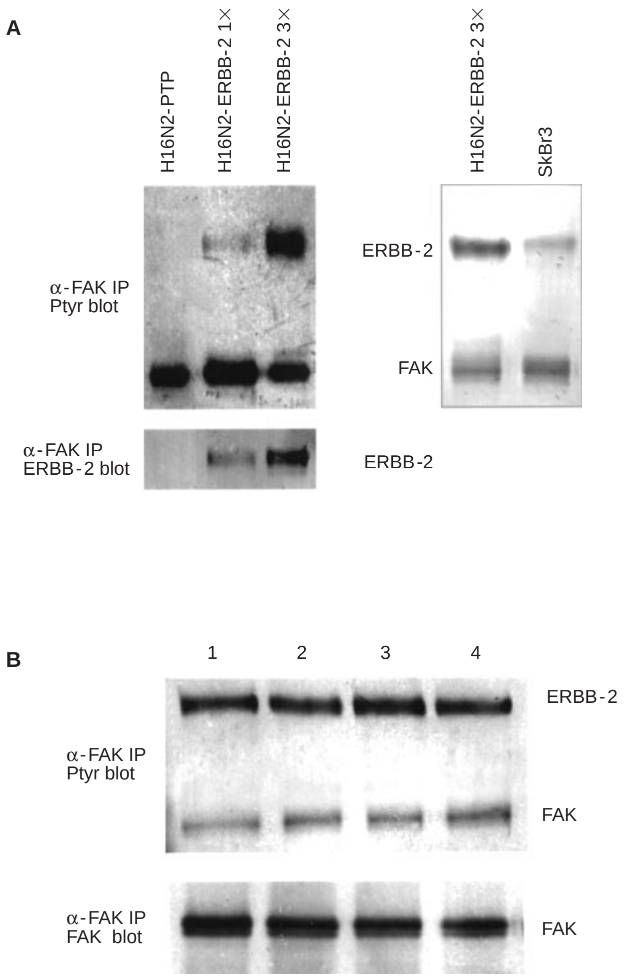

**Figure 5** ERBB-2 co-immunoprecipitates with FAK in H16N2-ERBB-2 cells. (**A**) Anti-FAK immunoprecipitates from control and ERBB-2 overexpressing cells probed with anti-PTYR$_{4G10}$. FAK and ERBB-2 are indicated; an identical blot was probed with anti-ERBB-2 to ensure that the tyrosine-phosphorylated p185 was ERBB-2. Anti-FAK immunoprecipitates from H16N2-ERBB-2 and SkBr-3 cells were also probed with anti-PTYR$_{4G10}$ to show co-immunoprecipitating tyrosine-phosphorylated p185. (**B**) FAK is not dephosphorylated in PTEN-expressing cells. Anti-FAK immunoprecipitations from H16N2-ERBB-2 (Lane 1), H16N2-ERBB-2/PTP (Lane 2), H16N2-ERBB-2/PTEN WT (Lane 3), or H16N2-ERBB-2/PTEN CS (Lane 4) cells were probed with anti-PTYR$_{4G10}$; an identical blot was probed with anti-FAK to show FAK levels

Table 1). These results, coupled with the data obtained from the experiments using LY294002, suggest that the ability of PTEN to block invasion of H16N2-ERBB2 cells is related to its ability to block PI 3′-kinase signalling rather than to its ability to directly dephosphorylate pp125$^{FAK}$. Indeed, our results are consistent with the previous observations (Maehama and Dixon, 1998) which indicate that under physiological conditions, PTEN does not dephosphorylate pp125$^{FAK}$. This finding raises the possibility that the interaction of p185$^{ERBB-2}$ with pp125$^{FAK}$ in ERBB-2 over-expressing cells can alter the expression or cellular localization of $\alpha_4\beta_1$ integrins. Further work will be required to dissect the roles of ERBB-2, PI 3′-kinase, and pp125$^{FAK}$ in the invasive potential of ERBB-2 overexpressing breast epithelial cells.

Taken together, the data obtained in these studies demonstrate that high-level overexpression of activated p185$^{ERBB-2}$ results in the acquisition of altered phenotypes typically expressed by aggressive cancer cells. ERBB-2 overexpressing cells are not only growth factor-independent and anchorage independent for growth, these cells can invade naturally occurring basement membranes in a pFn-dependent manner. When expressed to very high levels, p185$^{ERBB-2}$ can be detected in immunocomplexes with pp125$^{FAK}$, and blocking PI 3′-kinase signalling in these cells completely abrogates the invasive potential of H16N2-ERBB2 cells.

### DISCUSSION

ERBB-2 is an important oncogene in breast and other cancers, and overexpression and constitutive activation of its tyrosine kinase is essential to its oncogenic properties (DiFiore et al, 1987, 1990*a*, 1990*b*; Hudziak et al, 1987; Lonardo et al, 1990; Pierce et al, 1991). Despite the clear role of ERBB-2 in the aetiology of some human cancers, the specific cellular phenotypes expressed by cancer cells that are the direct result of ERBB-2 overexpression have not been completely defined.

In previous work, we demonstrated that breast cancer cells that overexpress p185$^{ERBB-2}$ are independent of growth factors required by normal HME cells for continuous proliferation in serum-free medium (Ram et al, 1996). More recently, we showed that over-expression of p185$^{ERBB-2}$ in HME cells to levels expressed by breast cancer cells with a ERBB-2 gene amplification is sufficient to induce the growth factor independence phenotype (Woods Ignatoski et al, 1999). The activation of signalling pathways, such as PI 3′-kinase and MAP kinase, in ERBB-2 overexpressing cells has been reported by a number of investigators and is consistent with the ability of the ERBB-2 kinase to stimulate growth of cancer cells (Benlevy et al, 1994; Marte et al, 1995). However, HBC cells exhibit other altered cellular phenotypes beyond growth factor independence. Therefore, we undertook experiments to determine if ERBB-2 overexpression in HME cells could induce other altered cellular phenotypes expressed by breast cancer cells.

H16N2 human mammary epithelial cells are a keratin-19-positive cell line that was immortalized by transduction of the entire HPV-16 genome (Band and Sager, 1989; Band et al, 1990). These cells are immortal in culture, are partially independent of insulin for growth in serum-free medium, but are still completely dependent on EGF for growth. In addition, H16N2 cells do not grow in soft agar, are not invasive in vitro, and do not form tumours in immunodeficient animals. H16N2-ERBB2 cells, which express levels of activated ERBB-2 equivalent to HBC cells with a ERBB-2 gene amplification, are independent of both insulin and EGF for growth in serum-free medium, have the ability to grow under anchorage-independent conditions, and can invade naturally occurring basement membranes. Thus, this is the first report to demonstrate that ERBB-2 overexpression can have a direct effect on the invasive potential of HME cells, and that progressive ERBB-2 overexpression results in the acquisition of phenotypes associated with increasing malignancy.

Based on the findings of our study, there are at least two pathways that are affected by overexpression of ERBB-2 which are important to the invasive potential of the cells. The first is the ERBB-2-mediated activation of PI 3′ kinase signalling. The second is the plasma fibronectin dependence for the invasive phenotype, and the related down-regulation of cell surface $\alpha_4$ integrin in the invasive cells.

Several laboratories, including our own, have demonstrated that overexpression of ERBB-2 results in high level activation of PI 3′

© 2000 Cancer Research Campaign 

kinase (Fedi et al, 1994; Soltoff et al, 1994; Ram and Ethier, 1996). There are at least three possible mechanisms by which ERBB-2 modulates the activity of this pathway (reviewed in Earp et al, 1986; Alroy and Yarden, 1997). First, the cytoplasmic domain of $p185^{ERBB-2}$ has a single YXXM binding site for the regulatory subunit of PI 3′ kinase. Thus, either direct or indirect phoshorylation of this tyrosine residue can result in activation of the enzyme. Second, ERBB-2 activation often results in the concomitant activation of ERBB-3, which has six YXXM sites in its cytoplasmic domain. Thus, ERBB-3 is a potent activator of PI 3′ kinase, and the H16N2-ERBB2 cells used in these studies express high levels of constitutively tyrosine phosphorylated ERBB-3. The third potential mechanism involves the association of the p85 subunit of PI 3′ kinase with tyrosine phosphorylated $pp125^{FAK}$ (Bachelot et al, 1996). This mechanism is potentially significant in our experiments since, in invasive ERBB-2 overexpressing cells, $p185^{ERBB-2}$ was found to be associated with activated $pp125^{FAK}$ in immunocomplexes, and the p85 subunit of PI 3′ kinase was detectable in the same immunocomplexes (not shown). Thus, it is possible that the association of $p185^{ERBB-2}$ with $pp125^{FAK}$ could potentiate PI 3′ kinase activation beyond what occurs as a result of ERBB-2 homodimer and ERBB-2/ERBB-3 heterodimer interactions. The data obtained in our studies indicate that PI 3′ kinase activation is a direct mediator of the invasive phenotype in that both LY294002 and exogenous expression of PTEN reversed the invasive capacity of H16N2-ERBB2 cells. The effect of PTEN is particularly interesting in that PTEN transduced cells proliferate as well in monolayer culture as control cells (data not shown). In contrast, the cells are growth inhibited by the concentration of LY204002 used to block invasion (data not shown).

The SU-basment membrane invasion assay used in these studies offers the ability to study cell invasion of naturally occurring basement membranes under serum-optional conditions. The defined nature of the assay allowed for the previous observation of the importance of plasma fibronectin for invasion of basement membranes by prostate cancer cells (Livant et al, 1995), and for the observation of its importance for basement membrane invasion by breast cancer cells, as described here. Like metastatic breast and prostate cancer cells, H16N2-ERBB2 cells also only invaded SU-basment membranes in the presence of plasma fibronectin. The importance of plasma fibronectin for invasion is particularly interesting because all cells examined thus far that are invasive in this assay have been found to express low levels of cell surface $\alpha_4$ integrin, while expressing high levels of $\alpha_5$. Indeed, cell surface $\alpha_4$ integrin expression, which is normal in H16N2 cells, was found to be down-regulated in ERBB-2 overexpressing cells with invasive potential. The down-regulation of $\alpha_4$ detected by FACs analysis was not observed in Western blots (not shown) suggesting that integrin trafficking is altered in the invasive cells. These results are consistent with those recently reported by Weaver et al (1997) who not only demonstrated that the repertoire of integrin expression is functionally important to the transformed phenotype of the cells, but that changes in cell-surface localization of integrins can occur without changes in overall levels of transcription or translation. Our data are also consistent with those of Seftor et al (1998) who demonstrated that maspin, which can modulate the invasive capacity of MDA-435 cells, can directly modulate cell surface expression of a number of integrins.

The loss of cell surface $\alpha_4\beta_1$ integrin in the presence of $\alpha_5\beta_1$ is important because each of these integrin dimers bind to distinct domains of plasma fibronectin. In normal cells, the cell binding domain of pFn interacts with $\alpha_5\beta_1$, whereas the C-terminal domain interacts with $\alpha_4\beta_1$ integrin. This dual interaction is important for the maintenance of normal cells in a non-motile, non-invasive state (Postlethwaite et al, 1981; Seppa et al, 1981; Clark et al, 1988). During wound healing, plasma fibronectin fragments are generated by proteolysis resulting in the formation of fragments containing the cell-binding domain. These fragments contain the PHSRN sequence, but do not contain the C-terminal domain that binds $\alpha_4\beta_1$ integrin. The stimulation of fibroblasts by proteolytic fragments of plasma fibronectin is essential for the invasion and migration of cells during this process (Grinnell et al, 1992). Indeed, Huhtala et al (1995), demonstrated that in fibroblasts, interaction of $\alpha_5\beta_1$ with pFn in the absence of $\alpha_4\beta_1$, results in the up-regulation of metalloproteinases that are important for invasion across basement membranes. Similarly, Livant et al (submitted for publication) demonstrated that direct stimulation of $\alpha_5\beta_1$ integrin using the PHSRN peptide derived from the pFn cell-binding domain, was sufficient to induce migration and invasion by normal epithelial cells. Thus, down-regulation of cell surface $\alpha_4$ integrin (and the consequent loss of $\alpha_4\beta_1$ plasma fibronectin receptors) in ERBB-2 overexpressing cells, as well as in breast and prostate cancer cells, is likely to be of functional significance to their ability to invade basement membranes and to metastasize.

Since integrins are involved in motility and invasion of cancer cells, and since integrin signalling is known to be mediated by activation of $pp125^{FAK}$ (Guan, 1997; Ilic et al, 1997), the observation that $p185^{ERBB-2}$ and $pp125^{FAK}$ interact and can be co-immunoprecipitated in invasive, ERBB-2 overexpressing cells, is particularly intriguing. This result suggests the possibility that the interaction of $p185^{ERBB-2}$ and $pp125^{FAK}$, and the down-regulation of $\alpha_4\beta_1$ integrin from the cell surface, are functionally related. In addition, since the p85 subunit of PI 3′ kinase is also detectable in $pp125^{FAK}$ immunoprecipitates, and blocking PI 3′ kinase signalling abrogates invasiveness of the cells, the interaction of $p185^{ERBB-2}$ and $pp125^{FAK}$ could be important for the overall activation of PI 3′-kinase signalling. Distinguishing the precise roles of $p185^{ERBB-2}$, $pp125^{FAK}$, PI 3′ kinase activation, and cell surface $\alpha_4\beta_1$ integrin expression in mediating the invasive phenotype will require further experiments.

In summary, the data reported here, and previously, indicate that progressive overexpression of ERBB-2 in HME cells results in the step-wise acquisition of malignant properties. Moderate ERBB-2 overexpression is sufficient to liberate cells from their requirements of exogenous insulin-like growth factors for proliferation, but is not sufficient to drive EGF independence, growth in soft-agar, or invasive capacity. However, expression of ERBB-2 to levels seen in breast cancer cells with a gene amplification not only results in complete growth factor autonomy, but also yields cells with robust anchorage-independent growth potential and with the ability to invade naturally occurring basement membranes. HME cells expressing high levels of $p185^{ERBB-2}$ exhibit many alterations in normal signalling patterns including; constitutive tyrosine phosphorylation of ERBB-2, ERBB-3 and EGFR, constitutive activation of PI 3′ kinase, association of ERBB-2 with $pp125^{FAK}$, and down-regulation of cell surface $\alpha_4\beta_1$ integrins. Future studies will be aimed at dissecting the relative contributions of each of these signalling pathways in mediating the different altered phenotypes exhibited by ERBB-2 overexpressing mammary epithelial cells and breast cancer cells.

 *© 2000 Cancer Research Campaign*

### ACKNOWLEDGEMENTS

We thank Cemal Sozener for excellent technical assistance, Amy Pace for help with the figures, and Dr Kim Orth for advice on experimental design and on the manuscript. This work was supported by NIH grant no. CA70354.

### REFERENCES

Akiyama SK and Yamada KM (1985) The interactions of plasma fibronectin with fibroblastic cells in suspension. *J Biol Chem* **260:** 4492–4500

Alroy I and Yarden Y (1997) The erbB signaling network in embryogenesis and oncogenesis: signal diversification through combinatorial ligan-receptor interactions. *FEBS Lett* **410**: 83–86

Anzick SL, Kononen J, Walker RL, Azorsa DO, Tanner MM, Guan X-Y, Sauter G, Kallioniemi O-P, Trent JM and Meltzer PS (1997) AIB1, a steroid receptor coactivator amplified in breast and ovarian cancer. *Science* **277**: 965–968

Aota S, Nomizu M and Yamada KM (1994) The short amino acid sequence Pro-His-Ser-Arg-Asn in human fibronectin enhances cell adhesive function. *J Biol Chem* **269**: 24756–24761

Bachelot C, Ramesh L, Parsons T and Cantley LC (1996) Association of phosphatidylinositol 3-kinase, via the SH2 domains of p85, with focal adhesion kinase in polymoma middle t-transformed fibroblasts. *Biochimica et Biophisica Acta* **1311**: 45–52

Band V and Sager R (1989) Distinctive traits of normal and tumor derived human mammary epithelial cells expressed in a medium that supports long-term growth of both cell types. *Proc Natl Acad Sci USA* **86**: 1249–1253

Band V, Zajchowski D, Swisshelm D, Trask D, Kulesa V, Cohen C, Connolly J and Sager R (1990) Tumor progression in four mammary epithelial cell lines derived from the same patient. *Cancer Res* **50**: 7351–7357

Benlevy R, Paterson HF, Marshall CJ and Yarden Y (1994) A single autophosphorylation site confers oncogenicity to the Neu/ErbB-2 receptor and enables coupling to the MAP kinase pathway. *EMBO J* **13**: 3302–3311

Berger MS, Locher GW, Saurer S, Gullick WJ, Waterfield MD, Groner B and Hynes NE (1988) Correlation of c-erbB-2 gene amplification and protein expression in human breast carcinoma with nodal status and nuclear grading. *Cancer Res* **48**: 1238–1243

Bieche I, Tomasetto C, Regnier CH, Moog-Lutz C, Rio M-C and Lidereau R (1996) Two distinct amplified regions at 17q11-q21 involved in human primary breast cancer. *Cancer Res* **56**: 3866–3890

Bonilla M, Ramirez M, Lopez-Cueto J and Gariglio P (1988) In vivo amplification and rearrangement of c-myc oncogene in human breast tumors. *J Natl Cancer Inst* **80**: 665–671

Borg A, Baldetorp B, Ferno M, Olsson H and Sigurdsson H (1992) *c-myc* amplification is an independent prognostic factor in postmenopausal breast cancer. *Int J Cancer* **51**: 687–691

Bradford MM (1976) A rapid and sensitive method for the quantitation of microgram quantities of protein utilizing the principle of protein dye-binding. *Analytical Biochemistry* **72**: 248–254

Clark RAF, Wikner NE, Doherty DE and Norris DA (1988) Cryptic chemotactic activity of fibronectin for human monocytes resides in the 120 kDa fibroblastic cell binding fragment. *J Biol Chem* **263**: 1215–12123

DiFiore PP, Pierce JH, Kraus MH, Segatto O, King CR and Aaronson SA (1987) erbB-2 is a potent oncogene when overexpressed in NIH/3T3 cells. *Science* **237**: 178–181

DiFiore PP, Segatto O, Leonardo F, Fazioli F, Pierce JH and Aaronson SA (1990*a*) The carboxy-terminal domains of erbB-2 and epidermal growth factor receptor exert different regulatory effects on intrinsic receptor tyrosine kinase function and transforming activity. *Mol Cell Biol* **10**: 2749–2756

DiFiore PP, Segatto O, Taylor WG, Aaronson SA and Pierce JH (1990*b*) EGF receptor and erbB-2 tyrosine kinase domains confer cell specificity for mitogenic signaling. *Science* **248**: 79–83

Earp HS, Austin KS, Blaisdell J, Rubin RA, Nelson KG, Lee LW and Grisham JW (1986) Epidermal growth factor (EGF) stimulates EGF receptor synthesis. *J Biol Chem* **261**: 4777–4780

Fedi P, Pierce JH, Difiore PP and Kraus MH (1994) Efficient coupling with phosphatidylinositol 3-kinase, but not phospholipase C gamma or GTPase-activating protein, distinguishes erbB-3 signaling from that of other erbB EGFr family members. *Mol Cell Biol* **14**: 492–500

Gillet CV, Fantl R, Smith C, Fisher J, Bartek C, Dickson D, Barnes and Peters G (1994) Amplification and overexpression of cyclinD1 in breast cancer detected by immunohistochemical staining. *Cancer Res* **54**: 1812–1817

Grinnell F, Ho CH and Wysocki AJ (1992) Degradation of fibronectin and vitronectin in chronic wound fluid: analysis by cell blotting, immunoblotting, and cell adhesion assays. *J Invest Derm* **98**: 410–416

Guan JL (1997) Role of focal adhesion kinase in integrin signaling. *Int J Biochem, and Cell Biol* **29**: 1085–1096

Guerin M, Gabillot M, Mathieu MC, Travagli JP, Spielmann NA and Riou G (1989) Structure and expression of c-erbB-2 and EGF receptor genes in inflammatory and non-inflammatory breast cancer: prognostic significance. *Int J Cancer* **43**: 201–208

Harris KF, Christensen JB, Radany EH and Imperiale MJ (1998) Novel mechanisms of E2F induction by BK virus large T antigen: requirement of both the pRb binding and J domains. *Mol Cell Biol* **18**: 1746–1756

Hayman EG, Pierschbacher M and Engvall E (1982) Fibronectin: purification, immunochemical properties, and biological activities. *Meth Enzymol* **82**: 803–831

Hudziak RM, Schlessinger J and Ullrich A (1987) Increased expression of the putative growth factor receptor p185Her2 causes transformation and tumorigenesis of NIH3T3 cells. *Proc Natl Acad Sci USA* **84**: 7159–7163

Huhtala P, Humphries MJ, McCarthy JB, Tremble PM, Werb Z and Damsky CH (1995) Cooperative signaling by α5β1 and α4β1 integrins regulates metalloproteinase gene expression in fibroblasts adhering to fibronectin. *J Cell Biol* **129**: 867–879

Ilic D, Damsky CH and Yamamoto T (1997) Focal adhesion kinase at the crossroads of signal transduction. *J Cell Science* **110**: 401–407

Jaakkola S, Salmikangas P, Nylund S, Partanen J, Armstrong E, Pyrhonen S, Lehtovirta P and Nevanlinna H (1993) Amplification of fgfr4 gene in human breast and gynecological cancers. *Int J Cancer* **54**: 378–382

Jacquemier J, Adelaide J, Parc P, Penaultllorca F, Planche J, Delapeyriere O and Birnbaum D (1994) Expression of the FGFR1 gene in human breast-carcinoma cells. *Int J Cancer* **59**: 373–378

Kraus MH, Popescu NC, Amsbaugh SC and King CR (1987) Overexpression of the EGF receptor-related proto-oncogene erbB-2 in human mammary tumor cell lines by different molecular mechanisms. *EMBO J* **6**: 605–610

Livant DL, Linn S, Markwart S and Shuster J (1995) Invasion of selectively permeable sea urchin embryo basement membranes by metastatic tumor cells, but not by their normal counterparts. *Cancer Res* **55**: 5085–5093

Lonardo F, DiMarco E, King CR, Pierce JH, Segatto O, Aaronson SA and DiFiori PP (1990) The normal erbB-2 product is a atypical receptor-like tyrosine kinase with constitutive activity in the absence of ligand. *New Biol* **2**: 992–1003

Luqmani YA, Graham M and Coombes RC (1992) Expression of basic fibroblast growth factor, FGFR1 and FGFR2 in normal and malignant human breast, and comparison with other normal tissues. *Br J Cancer* **66**: 273–280

Maehama T and Dixon JE (1998) The tumor suppressor, PTEN/MMAC1, dephosphorylates the lipid second messenger, phosphatidylinositol 3,4,5-trisphosphate. *J Biol Chem* **273**: 13375–13378

Marte BM, Grausporta D, Jeschke M, Fabbro D, Hynes NE and Taverna D (1995) NDF/heregulin activates MAP kinase and p70/p85 S6 kinase during proliferation or differentiation of mammary epithelial cells. *Oncogene* **10**: 167–175

Natali PG, Nocotra MR, Bigotti A, Venturo I, Slamon DJ, Fendly BM and Ullrich A (1990) Expression of the p185 encoded by Her2 oncogene in normal and transformed human tissues. *Int J Cancer* **45**: 457–461

Pierce JH, Arnstein P, DiMarco E, Artrip J, Kraus MH, Lonardo F, DiFiore PP and Aaronson SA (1991) Oncogenic potential of erbB-2 in human mammary epithelial cells. *Oncogene* **6**: 1189–1194

Postlethwaite AE, Keski-Oja J, Balian G and Kang A (1981) Induction of fibroblast chemotaxis by fibronectin: localization of the chemotactic region to a 140,000 molecular weight nongelatin binding fragment. *J Exp Med* **153**: 494–499

Ram TG, Dilts CA, Dziubinski ML, Pierce LJ and Ethier SP (1996) Insulin-like growth factor and epidermal growth factor independence in human mammary carcinoma cells with c-erbB-2 gene amplification and progressively elevated levels of tyrosine phosphorylated erbB-2. *Mol Carcinogen* **15**: 227–238

Ram TG and Ethier SP (1996) Phosphatidylinositol 3-kinase recruitment by p185 (erbB-2) and erbB-3 is potently induced by neu differentiation factor/heregulin during mitogenesis and is constitutively elevated in growth factor-independent breast carcinoma cells with c-erbB-2 gene amplification. *Cell Growth Differ* **7**: 551–561

Roklin OW and Cohen MB (1995) Expression of cellular adhesion molecules on human prostate tumor cell lines. *Prostate* **26**: 205–212

Sambrook J, Fritsch EF and Maniatis T (1989) *Molecular Cloning: A Laboratory Manual*, p18.45. Cold Spring Harbor Laboratory Press: Cold Spring Harbor.

Seftor RE, Seftor EA, Sheng S, Pemberton PA, Sager R and Hendrix MJC (1998) Maspin suppresses the invasive phenotype of human breast carcinoma. *Cancer Res* **58**: 5681–5685

© 2000 Cancer Research Campaign

Sen S, Zhou HY and White RA (1997) A putative serine/threonine kinase encoding gene BTAK on chromosome 20q13 is amplified and overexpressed in human breast cancer cell lines. *Oncogene* **14**: 2195–2200

Seppa HE, Yamada KM, Seppa ST, Silver MH, Kleinman HK and Schiffman E (1981) The cell binding fragment of fibronectin is chemotactic for fibroblasts. *Cell Biol Int Rep* **5**: 13–19

Slamon DJ, Clark GM, Wong SG, Levin WJ, Ullrich A and McGuire WL (1987) Human breast cancer: correlation of relapse and survival with amplification of the HER-2/neu oncogene. *Science* **235**: 177–182

Soltoff SP, Carraway KL, Prigent SA, Gullick WG and Cantley LC (1994) ErbB3 is involved in activation of phosphatidylinositol 3-kinase by epidermal growth factor. *Mol Cell Biol* **14**: 3550–3558

Tamura M, Gu J, Takino T and Yamada KM (1999) Tumor suppressor PTEN inhibition of cell invasion, migration, and growth: differnential involvement of focal adhesion kinase and p130Cas. *Cancer Res* **59**: 442–449

Watson PH, Safneck JR, Le K, Dubik D and Shiu RPC (1993) Relationship of c-myc amplification to progression of breast cancer from in situ to invasive tumor and lymph node metastasis. *J Natl Cancer Inst* **85**: 902–907

Weaver VM, Petersen OW, Wang F, Larabell CA, Briand P, Damsky C and Bissell MJ (1997) Reversion of the malignant phenotype of human breast cells in three-dimensional culture and in vivo by integrin blocking antibodies. *J Cell Biol* **137**: 231–245

Witkowski CM, Rabinovitz I, Nagle RB, Affinito KD and Cress AE (1993) Characterization of integrin subunits, cellular adhesion and tumorigenicity of four human prostate cell lines. *Cancer Res Clin Oncol* **119**: 637–644

Woods Ignatoski KM, LaPointe AJ, Radany EH and Ethier SP (1999) ErbB-2 overexpression in human mammary epithelial cells confers growth factor independence. *Endocrinology* **140**:3615–3622

 *© 2000 Cancer Research Campaign*
